# Educational Intervention and Menopausal Adapting: A Randomized Controlled Trial

**DOI:** 10.1002/hsr2.70298

**Published:** 2024-12-29

**Authors:** Mansooreh Khandehroo, Maryam Salary, Mehrsadat Mahdizadeh, Nooshin Peyman

**Affiliations:** ^1^ Department of Health Education and Health Promotion, Social Determinants of Health Research Center, School of Health Mashhad University of Medical Sciences Mashhad Iran; ^2^ Department of Epidemiology, Social Determinants of Health Research Center, Faculty of Epidemiology Mashhad University of Medical Sciences Mashhad Iran

**Keywords:** adaptation, educational intervention, menopause

## Abstract

**Background and Aims:**

Menopause is a multifaceted condition with several problems. Educational interventions regarding self‐care and limiting exposure to menopausal warning indicators can help to minimize problems. As a result, the study aimed to investigate the influence of educational intervention on variables influencing menopausal adaptation.

**Methods:**

This study was a randomized controlled trial (RCT) intervention in which the researcher developed a standard questionnaire (kapa = o/99, CVR = 0/81, CVI = 0/96, ICC = 0/89) for 130 participants at four education sessions in Mashhad, Iran, in 2023. This questionnaire was completed before the intervention, shortly afterward, and 3 months later.

**Results:**

The average age was 52 ± 4 years, with no significant difference between the two groups. According to the regression model findings immediately following the intervention, the factors of education level, income level, and age had no significant influence on the overall score of the questionnaire. The linear regression model revealed that the intervention group had a higher average score of 13.89. The outcomes 3 months after the intervention were identical to those immediately following the intervention. Three months after the intervention, there was a substantial change in the score for encountering warning signals and seeking help and self‐care compared to before (*p*‐value = 0.002, *p*‐value < 0.001, *p*‐value = 0.003).

**Conclusion:**

According to the findings of this study, the educational intervention improved the adjusting variables to menopause. Designing proper education interventions can improve menopause adaption by increasing the element of seeking support and self‐care, as well as coping with menopausal warning symptoms. Longer interventions, however, are required to influence menopausal appraisal and comprehension.

## 1 Introduction

Menopause is a physiological event that affects all women worldwide. It is a dynamic and evolutionary process that lasts approximately one‐third of a woman's life [1]. However, the experience of menopause varies from person to person. In a study by Peyman et al. [2], one group saw menopause as the start of old age, while another saw it as the start of getting rid. People who felt liberated responded better to menopause and experienced less uncomfortable symptoms. These individuals have improved physical, mental, and social situations. Hong Kong research verified this finding [3].

In Australia, persons with a positive attitude reported fewer symptoms, had less stress, and dealt with menopause better [4]. According to Aaron and colleagues, women's reactions to menopause are determined by their attitudes regarding it [5]. One of the key concepts of menopausal adaptation, according to a study of Taiwanese women, was accepting the changes induced by menopause and the associated identity shift [6].

According to Lia et al., persons who recognized menopause as a natural part of life adapted more quickly [7].

The perceived sensations include the experience of being worthless [8], depression, despair [9], the feeling of being ugly [10], reaching the end of the journey [11], amazement, and the feminine end feeling [11]. Women's perceptions of menopause are multifaceted, stemming from bodily changes, societal influences, and sentiments that are unique to each person [2].

In this research, adapting to menopause means a positive reaction to this change, not giving in to the complications of menopause, and proper interaction with society and the environment.

Because of the significance of adapting to menopause, this intervention aimed to investigate the effect of the educational intervention on the adoption of menopause.

## 2 Methods

This interventional study, an RCT (randomized controlled trial), included a control group. The IRCT, ID is 54998. The participants were menopausal women who visited health clinics in Mashhad, Iran, in 2023. Because there were no comparable papers, the sample size was calculated using the effect size.

Given the average impact size, this will be equal to 0.5. The sample size for each group was 65, assuming 80% test power and a 5% starting error. Sampling was conducted using a randomized technique.

The permutation random blocks method is used to randomly assign the treatments to the study subjects while ensuring equal numbers in the study groups. For this purpose, blocks of sizes 2 and 4 are used, and a random list is created through the website https://www.sealedenvelope.com. For example, the random list can be [A B] [A A B B] [B A].

Inclusion criteria: declaration of permission to participate in the study, women aged 45–65 with menopausal experience (Amenorrhea for more than 1 year), literacy, Iranian nationality, and Persian proficiency. Exclusion criteria include a reluctance to continue engaging in the intervention, and diabetic, hypertension.

Demographic characteristics included age, education, wealth or poverty, social position, employment, and social group participation. Demographic variables have been homogenized in both test and control groups.

The control group had a suitable geographic distance from the test group. There was no relationship between the test and control groups. The control group was not trained. After the research was finished and the data analyzed, the control group was also trained. According to the article, Health Literacy Intervention and quality of life in menopausal women: a randomized controlled [2], the period of intervention has been effective in creating significant change thus, for the test group, the intervention included four education sessions separated by a 1‐week intervals.

### 2.1 Educational Intervention Process

**Figure 1:**
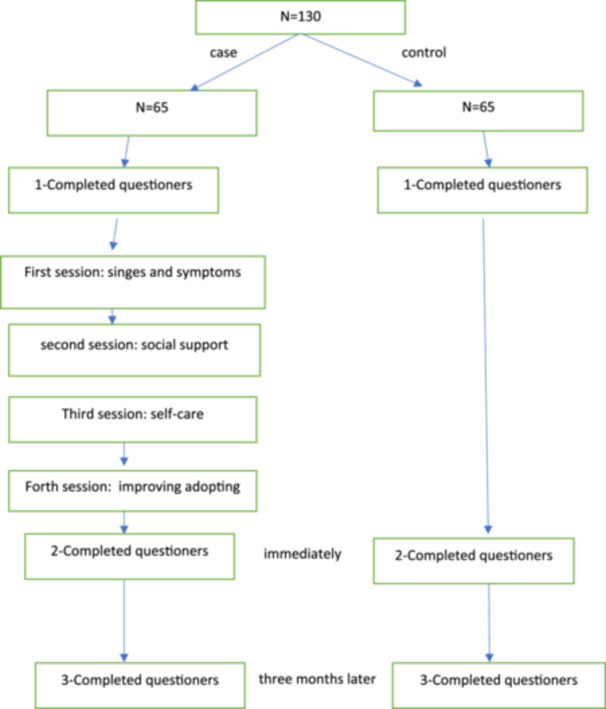
Intervention diagram.

Menopausal adoption is a main outcome that has been measured by four variables: Evaluation and understanding of menopause, encountering menopausal signs and symptoms, Seeking support, and Self‐care. Four education sessions took place face‐to‐face 45‐min. The first lesson focused on warning indications of menopause, such as hot flashes and discomfort. Some of these symptoms are common with age, and all affect menopausal health. The second session addressed social support. Social support influences menopausal experiences and has an important role in minimizing menopausal symptoms. In this session, we discussed how they can attract more love, help, and attention from family members, friends, and others associated with the individual. Stress exacerbated women's dependence on the family during menopause. The third session focused on self‐care in menopause. Lack of knowledge about menopause can sometimes lead to nonacceptance and the onset of psycho‐emotional symptoms in the post‐menopausal period. We stated that adopting can increase your capacity to regulate impulses and emotions. The final session focused on an improved attitude, a healthier lifestyle, increased knowledge, and participation in social contacts. These elements can aid with menopausal adoption. Participants completed the questionnaire before, immediately, and 3 months afterward. The data were then used to assess the effects of educational interventions on menopausal adaptation factors. This process was written in Figure 1.

The questionnaire utilized was a researcher‐created questionnaire on menopausal adaption. The researcher conducted a psychometric evaluation of this questionnaire and found good validity, reliability, and validity (kappa = o.99, CVR = 0.81, CVI = 0.96, ICC = 0.89). This standardized questionnaire has 46 items. The questions are scored on a 5‐point Likert scale (totally agree (5), agree (4), have no opinion (3), disagree (2), and completely disagree (1)). The questions 1, 2, 5, 6, 7, 9, 10, 11, 12, 29, and 30 were scored inversely. The overall score is 230, with the cutoff scores of 76 and 152.

The data were analyzed and described using frequency distribution tables, graphs, central tendency indices (mean, median, and mode), and dispersion indices (variance, standard deviation, coefficient of variation, and range of variation.

Confounder variables were corrected using statistical testing, regression, and covariance analysis. Values are presented as mean ± standard deviation. Following the normality tests by conducting the Shapiro‐Wilk tests, the Chi‐square test was used to evaluate the association between qualitative variables, the *t*‐test to compare mean values in normal variables, and the Mann‐Whitney test in non‐normal variables. The correlation coefficient was used to evaluate the pairwise correlation of variables and multiple linear regression and generalized estimating equity (GEE) test were used to evaluate the relationship between dependent and independent variables. Essentially GEE models extend generalized linear models for the situation of correlated data. Thus, this class of models has become very popular, especially in the analysis of categorical and count outcomes, though they can used for continuous outcomes as well. GEE models are termed marginal models, and they model the regression of y on X and within‐subject dependence, this term marginal in this context indicates that the model for the mean response depends only on the covariates of interest, and not on any random effects or previous responses.

The significance level was 0.05 in all cases. We did all the tests in two domains because the significant increase and significant decrease were important to us.

We adhered to the SAMPL guidelines and followed the CONSORT guidelines when revising the manuscript. The software used for quantitative data were SPSS version 16.

The data were then used to assess the effects of educational interventions on menopausal adaptation factors. Adopting is a main outcome that has been measured by four variables: Evaluation and understanding of menopause, encountering menopausal signs and symptoms, seeking support, and self‐care. Finally, the effects of educational interventions on menopausal adaption variables were evaluated based on the findings.

## 3 Results

The research included 130 individuals who were randomly allocated to either the intervention or control groups. After conducting the Shapiro‐Wilk tests and finding that all variables were non‐normal, we proceeded with the Mann‐Whitney test, multiple linear regression test, and the GEE test, all at a significance level of 0.05. The GEE model showed a significant confounding impact of time (*p*‐value < 0.001) and group and time (*p*‐value < 0.001). Each questionnaire was independently entered into the univariate regression model and checked at the desired three times. Then, we reported these results. In our study, the level of education was not significant in adaptation to menopause. We honestly reported that this may be a little far from reality. Fortunately, it can be said that education is done properly and has a positive effect although it isn't significant. The age variable did not differ substantially between the control and intervention groups (*p*‐value < 0.001). Table 1 displays the demographic factors for this investigation.

**Table 1:** Demographic variables of participants.

Variable	Value	Frequency (%)	Variable	Value	Frequency (%)
**Number child**	None	(%5/38)7	Education	Bachelor's degrees	
	One	(%40)52			(%31/51)41
	Two and more	(%54/61)71		Diploma degrees	(%68/49)89
**Job**	Housewife	(%79)103			
	Employee	(%6/92)9			
	Freelance	(%6/15)8			
	Retired	(%7/69)10			

The test and control groups' results were determined at each step as a total score for each participant and separately for each component. Table 2 demonstrates the normality of the variables.

**Table 2:** Normality of variable.

Variable	*P* (Shapiro–Wilk)
**Job**	0/03
**Number child**	0/01
**Education**	0/04
**Evaluating**	0/01
**Warning signs**	0/03
**Selfcare**	0/04
**Seeking support**	0/02

Table 3 displays the results. The GEE model was used to assess the effect of the intervention on confounding factors resulting from longitudinal data, as well as the interaction between time and group. At each stage of the analysis, a single‐variable regression was used. In this study, the intervention resulted in a substantial rise in total scores obtained immediately and 3 months later (confidence interval: 4.51–23.98, *p*‐value ≈ 0.001) in the test group.

**Table 3:** Scores obtained by the test and control group, in each period.

Factor	The mean and standard deviation before the intervention		*P* (Mann–Whitny)	The mean and standard deviation immidiatly		*P* Mann–Whitny)	The mean and standard deviation three months after the intervention		*P* Mann–Whitny)
	Control	Test		Control	Test		Control	Test	
Evaluating	32.7 ± 2.98	18.96 ± 4.26	< 0.001	28 ± 2.97	25.18 ± 2.94	< 0.001	32.79 ± 3.01	29.26 ± 4.14	< 0.001
Warning signs	57.39 ± 0.69	36.46 ± 0.99	< 0.001	57.39 ± 0/69	55.58 ± 0.87	0/145	57.06 ± 0.70	59.1 ± 0.77	0.04
Selfcare	55.25 ± 0.69	35.43 ± 1.19	< 0.001	55.25 ± 0.69	54.06 ± 0.79	0.149	55.13 ± 0.69	58.01 ± 0.56	0.151
Seeking support	42.83 ± 0.49	28.03 ± 0.85	< 0.001	42.83 ± 0.49	41.78 ± 0.65	0.36	42.69 ± 0.50	43.93 ± 0.52	≅0.001
Total	188.17 ± 1.87	118.88 ± 7.29	< 0.001	183.47 ± 4.8	176.6 ± 5.25		187.67 ± 1.89	190.3 ± 5.99	≅0.001

There was a significant difference between the test and control groups at all three phases (*p* ≈ 0.001). The confounding factor was controlled using the GEE test (*p*‐value ≈ 0.01). The GEE model revealed a significant confounding impact of time (*p*‐value < 0.001) and group and time (*p*‐value < 0.001). As a result, the findings were validated individually for each group using the univariate regression model.

There was no significant difference between the test and control groups in the evaluation and comprehension of menopausal scores because the group's regression index was insignificant in Table 4 (*p*‐value = 0.176) immediately after the intervention. Table 5 shows a similar result 3 months following the intervention (*p*‐value = 0.376). Table 6 shows the regression model on the total score of the questionnaire 3 months after the intervention. The educational intervention had a significant effect immediately and 3 months later on the encountering menopause symptoms and seeking support and self‐care based on the regression test. This is reported in 5 and 6 tables.

## 4 Discussion

According to the current study, the educational intervention had a good influence on the menopausal adaptation variables. The educational intervention had a significant effect immediately and 3 months later on the encountering menopause symptoms and seeking support and self‐care. It also resulted in the proper identification of menopausal warning symptoms [12, 13].

This intervention resulted in a problem‐based confrontation with menopause and a reduction in the intensity of their emotional exposure. Using emotional coping strategies is an inappropriate method to deal with stressful events. If problem‐solving abilities improve, we will be able to adapt more effectively [14].

According to an American's study, women who used problem‐based coping methods had better physical and mental health than women who used emotion‐based coping strategies [15]. It was also proven in the current research. Furthermore, the regression indexes in Tables 3 and 4 show that the present educational intervention had a favorable influence on seeking support and self‐care, adaption.

**Table 4:** The regression model on each factor immediately after the intervention.

	Variable		Regression	*P*	Confidence interval	
					Lower	Upper
Evaluating	Age		0.013	0.475	−0.067	0.093
	The score before intervention		0.328	< 0.001	0.196	.461
	Group	Test	1.44	0.167	−3.493	0.610
		Control				
	Education	Diploma degree	1.056	0.126	−0.03	2.413
		Bachelor's degrees	Reference			
Warning signs	Age		−0.35	0.693	−0.211	0.14
	The score before intervention		0.319	< 0.001	0.162	.476
	Group	Test	5.338	0.007	1.462	9.214
		Control				
	Education	Diploma degree	1.971	0.195	−1.23	4.964
		Bachelor's degrees	Reference			
Selfcare	Age		0.12	0.884	−0.153	0.177
	The score before intervention		0.293	< 0.001	0.165	.420
	Group	Test	4.556	0.006	1.355	7.760
		Control	Reference			
	Education	Diploma degree	1.617	0.26	−1.214	4.448
		Bachelor's degrees	Reference			
Seeking support	Age		−0.035	0.588	−0.161	0.091
	The score before intervention		0.317	< 0.001	0.182	.451
	Group	Test	3.594	0.005	1.091	6.97
		Control	Reference			
	Education	Diploma degree	1.703	0.12	−0.449	3.856
		Bachelor's degrees	Reference			

**Table 5:** The regression model of each factor in 3 months after the intervention.

	Variable		Regression	*P*	Confidence interval	
					Lower	Lower
Evaluating	Age			0.734	−0.083	0.117
	The score before intervention			< 0.001	0.178	0.511
	Group	Test	1.161	0.376	−3.744	1.423
		Control	Reference			
	Education	Diploma degree	0.774	0.372	−0.934	2.482
		Bachelor's degrees	Reference			
Warning signs	Age			0.420	−0.101	0.241
	The score before intervention			0.034	0.013	0.319
	Group	Test	5.882	0.003	2.102	9.662
		Control				
	Education	Diploma degree	−1.12	0.449	−4.039	1.8
		Bachelor's degrees	Reference			
Selfcare	Age			0.786	−0.123	0.162
	The score before intervention			< 0.001	0.99	0.319
	Group	Test	7.101	< 0.001	4.341	9.86
		Control				
	Education	Diploma degree	−1.006	0.416	−3.44	1.432
		Bachelor's degrees	Reference			
Seeking support	Age			0.637	−0.088	0.143
	The score before intervention			< 0.001	0.111	0.362
	Group	Test	4.82	< 0.001	2.488	7.152
		Control	Reference			
	Education	Diploma degree	−0.282	0.777	−2.247	1.684
		Bachelor's degrees				

**Table 6:** The regression model on the total score of the questionnaire 3 months after the intervention.

	Variable		Regression	P	Confidence interval	
					Lower	Lower
Total score after 3 months intervention	Age		0.152	0.432	−0.229	0.532
	Total score before intervention		0.159	0.011	0.037	0.282
	Group	Test	14.247	0.004	4.514	23.981
		Control	Reference			
	Education	Diploma degree	−1.720	0.602	−8.228	4.789
		Bachelor's degrees	Reference			

Despite the favorable outcomes of this intervention, there was no significant difference in assessment and comprehension scores between the test and control groups immediately, and 3 months after the intervention according to the regression index of the group in Table 5 (*p*‐value = 0.167), Table 6 (*p*‐value = 0.376).

Research has shown that menopausal women often suffer from a lack of information, and increasing their level of awareness of the problems and experiences of menopause will improve the quality of life during this period. How women understand the phenomenon of menopause affects their compatibility with the experiences of this era. According to the opinion of researchers, all women can improve their quality of life in this period by being aware, recognizing the experiences, and problems of menopause, and learning strategies to adapt to them [16]. If middle‐aged women do not go through this period with health, they will face double problems in their old age. In addition to raising personal issues for themselves, it also imposes a lot of problems and costs on society at the macro level.

The best action in this regard is to plan for women's health from the ages before old age, that is, youth and middle age. The results of some studies indicate that sometimes social contexts that shape women's perception and experience of body and health can create social, cultural, and mental obstacles to the success of health‐oriented programs and successful aging.

Therefore, to attract women's attention and their cooperation in health‐oriented programs, it is necessary to first change these social and mental beliefs and formative platforms so that women actively and responsibly participate in health plans [16]. Menopause might be followed by the emergence of chronic disorders such as hypertension and diabetes [14]. Thus, self‐care includes a series of habits and behaviors that we do consciously and purposefully to live healthier and happier and help ourselves to experience a higher quality of life, and be able to improve our physical, psychological, social, and spiritual needs, and experience a balanced life. Communication is an important tool for creating external balance and an effective way to eliminate internal disturbances. Having dynamic and warm relationships with positive people can help you develop your communication skills.

In this research, postmenopausal women integrated action‐based self‐care practices into their everyday routines. Some self‐care activities include eating a healthy diet rich in fruits and vegetables, legumes, healthy oils, and fish oil, avoiding sugar, caffeine, and processed foods, engaging in exercise and physical activity, and undergoing annual medical tests such as pap smears, mammography, bone densitometry, cholesterol tests, blood sugar levels, thyroid tests, and exercise tests.

These self‐care exercises helped alleviate menopausal symptoms. This endeavor enabled women to live easily and with more compatibility. The research show that women who have useful jobs during menopause feel more powerful and authoritative are lively and full of life and feel their femininity and completeness.

Scientific findings show that women who take on many different roles in life and maintain many social relationships are healthier and live longer than other women.

Extensive social connections not only destroy the factors that threaten loneliness and isolation but also boost self‐confidence and are highly beneficial for health [17].

In the phenomenon of menopause, full understanding and full acceptance of passing from the youth stage and entering a new stage of life will reduce complications and annoying problems improve the quality of life, and increase adaptation to menopause in the rest of life. The educational intervention has been able to deal more correctly with the warning signs of menopause. It helped women to deal with menopause in a problem‐oriented way and the intensity of their emotion‐oriented approach was reduced. Correcting exposure to menopause warning signs increases the level of adaptation of a person to menopause.

Menopause, as an acceptance of the termination of femininity, deepens social connections. The change of feelings following menopause is the factor of change in the way of interactions and the factor of humanism and social sympathy. For this reason, having the right knowledge and attitude towards the changes of this era can help a lot to coordinate with these changes.

This research demonstrates that the educational intervention did not affect people's perceptions or understanding of menopause, because early (even preverbal) experiences, affective experiences, cultural biases, and cognitive consistency principles influence on attitudes.

The menopausal adaption process necessitates a comprehensive understanding of the concept of adaptation, influencing variables, and mutual influence. It is a multifaceted process influenced by physical, psychological, behavioral, cognitive, environmental, and social elements [18]. The effect will be more stable if it is accompanied by a reminder. But in our study, due to the limitations, this issue was not predicted. As a result, it proposes that researchers use this comprehensive strategy in future studies. We suggest that future studies focus more on family support and societal issues. We attempted by adoption of the guidelines increase the quality of manuscript and improve statistical knowledge in our field in general [19].

### 4.1 Limitation

One of the limitations of this study was that it is not possible to say definitively whether the symptoms of menopausal women are related to aging or menopause. Another limitation of this study was that one of the variables investigated was the attitude. Sometimes social contexts shape women's attitudes and experience of health. Improvement of social, cultural, and mental obstacles is very difficult, and cannot be altered in this short period.

The strength of this study is that the equations derived may be used to predict the outcome of future educational activities. This endeavor helps women adjust to this period of life and live more comfortably and worry‐free.

## Author Contributions

**Mansooreh Khandehroo:** conceptualization, investigation, writing–original draft, writing–review and editing, methodology, visualization, software, formal analysis, project administration, data curation, resources. **Maryam Salary:** methodology, validation, formal analysis, visualization. **Mehrsadat Mahdizadeh:** investigation, supervision, data curation, project administration. **Nooshin Peyman:** supervision, conceptualization, project administration.

## Ethics Statement

All procedures performed in studies were by the ethical standards of the institutional research committee with the 1964 Helsinki Declaration. This proposal has been approved by the Ethics Committee of Mashhad University of Medical Sciences; the ethics code IR.MUMS.FHMPM.REC.1400.076. Address: https://ethics.research.ac.ir/IR.MUMS.FHMPM.REC.1400.076.

## Conflicts of Interest

The authors declare no conflicts of interest.

## Transparency Statement

The lead author Nooshin Peyman affirms that this manuscript is an honest, accurate, and transparent account of the study being reported; that no important aspects of the study have been omitted; and that any discrepancies from the study as planned (and, if relevant, registered) have been explained.

## Supporting information

Supporting information.

## Data Availability

Data sharing not applicable to this article as no datasets were generated or analysed during the current study. All data generated or analyzed during this study is included in this published article. All authors have read and approved the final version of the manuscript [CORRESPONDING AUTHOR or MANUSCRIPT GUARANTOR] had full access to all of the data in this study and took complete responsibility for the integrity of the data and the accuracy of the data analysis.
